# Ten simple rules for designing analogue science games

**DOI:** 10.1371/journal.pcbi.1009009

**Published:** 2021-06-10

**Authors:** Sam Illingworth, Paul Wake

**Affiliations:** 1 Department of Learning and Teaching Enhancement, Edinburgh Napier University, Edinburgh, United Kingdom; 2 Department of English, Manchester Metropolitan University, Manchester, United Kingdom; Dassault Systemes BIOVIA, UNITED STATES

## Introduction

Effective communication between scientists and nonscientists is essential to create a more inclusive society through better science engagement and participation [1]. Two of the biggest challenges facing effective science communication lie in determining your audience and deciding upon the mode through which this communication will take place [2]. In order for science communication to be more effective, it is necessary to consider which audience (or audiences) you wish to engage with and why, eschewing the ideal of an amorphous “general” public which does not really exist [3]. With regard to the mode of communication, much has been written about the need to move away from a deficit model of knowledge exchange (in which scientists convey information to nonscientists in a one-directional manner, filling their knowledge “deficit”) towards one of dialogue and participation [4].

In considering how best to develop such communications, a variety of media, ranging from poetry [5] and dance [6] to theatre [7] and comedy [8], have been explored. Similarly, research has been dedicated to the role that games might play in communicating scientific topics [9–12], including useful advice for game designers seeking to embed scientific endeavour within serious scientific games [13]. However, this research has tended to focus on digital games, neglecting the unique affordances of analogue games. Building on this research, this article is concerned with the design of analogue games (e.g., card, dice, and board games) for science communication.

Analogue games have several features that make them well suited to effective science communication [14]. These include their material properties (which can be used to engage participants in a variety of ways), their ease of modification (which can empower both designers and players), and their capacity to bring people together via face-to-face interactions. The quality of these face-to-face interactions is crucial: in negotiating the contracts of what is “acceptable” in the games that we play, we step away from hierarchies that might exist away from the gaming table, facilitating interactions that might not otherwise be possible. For example, when playing *King of Tokyo* [15] (a board game in which players take on the role of monsters fighting to control the city), it is perfectly permissible, and indeed advisable, to destroy opponents with poison spit, psychic probes, and shrink rays. We argue that the adoption of the appropriate lusory attitude [16] (the agreement to play by a set of arbitrary and often limiting rules), supported by a game’s physical “props” [17], can be leveraged to create a safe space in which new interactions and learning might take place, a space that suspends, albeit temporarily, hierarchies of knowledge [18].

These opportunities for lusory dialogue mean that analogue games offer scientific researchers a unique and versatile means by which to engage with a variety of nonscientific audiences. Furthermore, analogue science games offer the opportunity not only for effective science communication of a specific scientific topic but also for opening up dialogue about science itself, including topics relating to diversity, trust, and ethical practices. However, these engagements will ultimately only be as successful as the game itself: poorly designed analogue science games run the risk of putting audiences off both games and science altogether.

Based on our experiences as game designers and educators, we now set out 10 rules for designing analogue science games.

### Rule 1: Identify your topic

The first thing to determine is what it is that you want to communicate. This discussion must come before you can determine the appropriate mode of communication.

In the Introduction, we noted 2 seemingly opposed methods that you might wish to consider: deficit and dialogue. In the most basic terms, these 2 methods might suggest 2 different types of games: those that aim to communicate facts and those that aim to start conversations. While dialogue is widely accepted to be the preferred method of science communication, we recognise that certain objectives might be best served by some form of dissemination and that providing reliable information in an accessible way is often an essential prerequisite for dialogue to occur [19]. Accordingly, in what follows, we set aside the term “deficit model,” which holds negative implications about both the intellectual capacity of an audience and the potential motivations of scientists [20–22], in favour of “dissemination” (by which we mean the sometimes necessary approach of conveying information in a one-way direction) and recognise that in truth, most games will lie somewhere on the spectrum of “dissemination” to “dialogue.”

The scientific topic and the audience that you wish to engage with (Rule 2) will largely determine where you want your game to lie on this spectrum. For example, you might want to design a game that serves to introduce your audience to different types of plant phenomics so that they might be better informed for conducting an image analysis task [23], in which case you might concentrate on dissemination. Conversely, if you were designing a game to raise awareness of the ethical challenges of big data in public health [24], then a focus on initiating and sustaining dialogue might be more appropriate.

**Fig 1:**
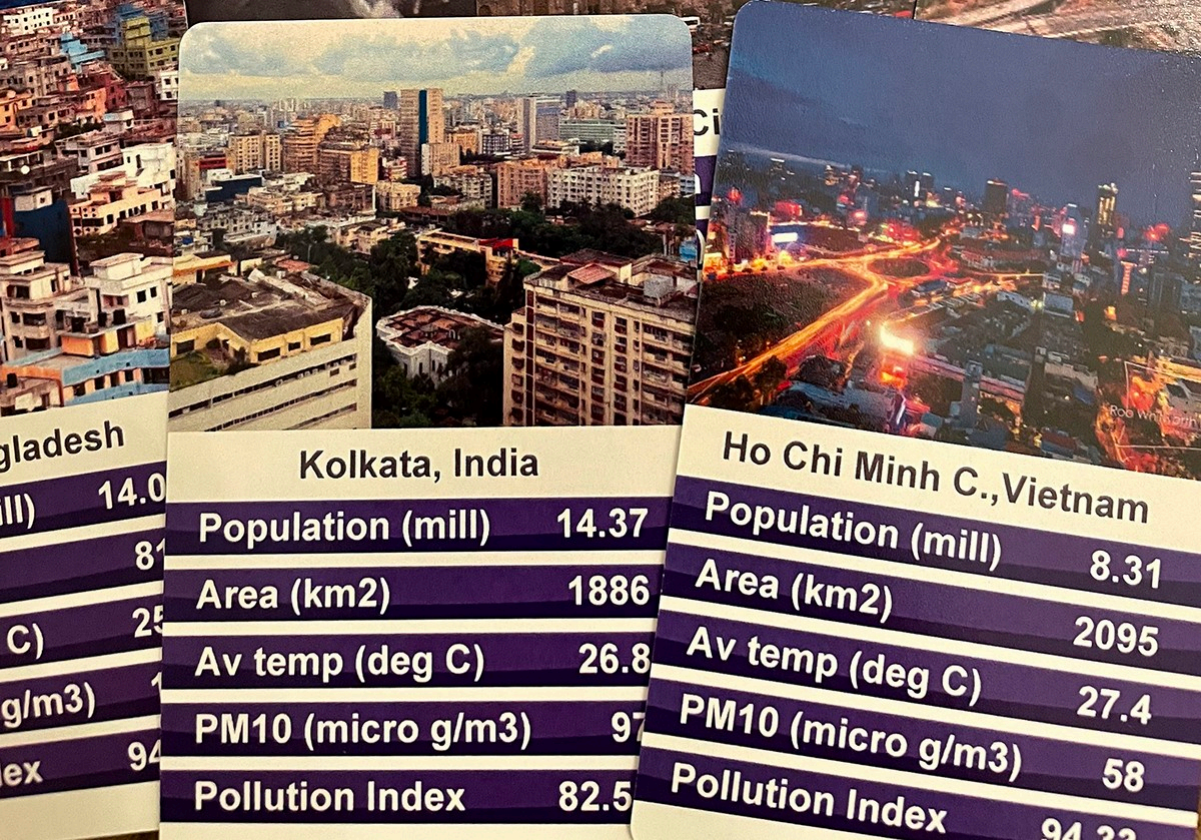
A game that was designed to communicate information about the pollution index of the world’s 50 most polluted cities, based on *Top Trumps*.

To give a specific example, Fig 1 shows a card game that was designed by one of the authors as part of a science communication initiative to communicate facts relating to the 50 most polluted cities in the world.

In this game (based on *Top Trumps*), the object is to collect all of the cards in the deck by comparing their statistics, and the player with the highest value each round claims the cards. In playing this game, players learn about the extent of the pollution in these cities, and as such, it clearly places an emphasis on the dissemination of information. However, observing groups playing the game revealed that it also worked to initiate dialogue (“I had no idea Osaka was so polluted,” “I really think that Tehran should trump London,” “Where did they get these measurements from?”). In this specific example, the relative simplicity (and familiarity) of the game makes it possible for players to engage in gameplay and discussion simultaneously, with the game providing visual and textual prompts to scaffold both activities.

### Rule 2: Identify your audience

The appropriate position of your game on the dissemination-to-dialogue spectrum will in part be determined by the needs and experiences of your intended audience(s). As we discussed in the Introduction, there is no such thing as a general public rather, there are many publics [25], and each of them will have their own barriers and opportunities for engagement, as well as their reasons for wanting to engage (or not) with science. In determining which public you want to engage, begin by giving proper consideration to both what and why you are trying to communicate and why it is that you wish to interact with this audience in particular [2].

Once you have determined why you want to engage with a particular audience, consider the constraints for engagement and use these to construct a basic set of design parameters. For example, if your aim is to design a game about acoustic ecology [26] for schoolchildren in a classroom setting, then you are limited by several factors, namely space (the size of classrooms and of classroom tables), time (the duration of a lesson), and scientific literacy (think what these students know already about acoustic ecology, what you would like them to find out as a result of playing your game, and to what extent you can rely on a teacher to supplement any knowledge). At this stage, you may want to revisit Rule 1 and think again where on the dissemination-to-dialogue spectrum your game needs to lie.

It is also important to consider the game playing experience of your audience. What games do they already play? With the answer to this question in mind, ask yourself, how complex can you make your game without it becoming alienating? If you are an avid gamer, then this is something to be particularly careful of. Just as scientists need to be mindful that their everyday language might not readily be understood by nonspecialists, regular gamers should cultivate an awareness that an easy game for them to “pick up and play” might require careful explanation for those who play analogue games only occasionally, if at all.

**Fig 2:**
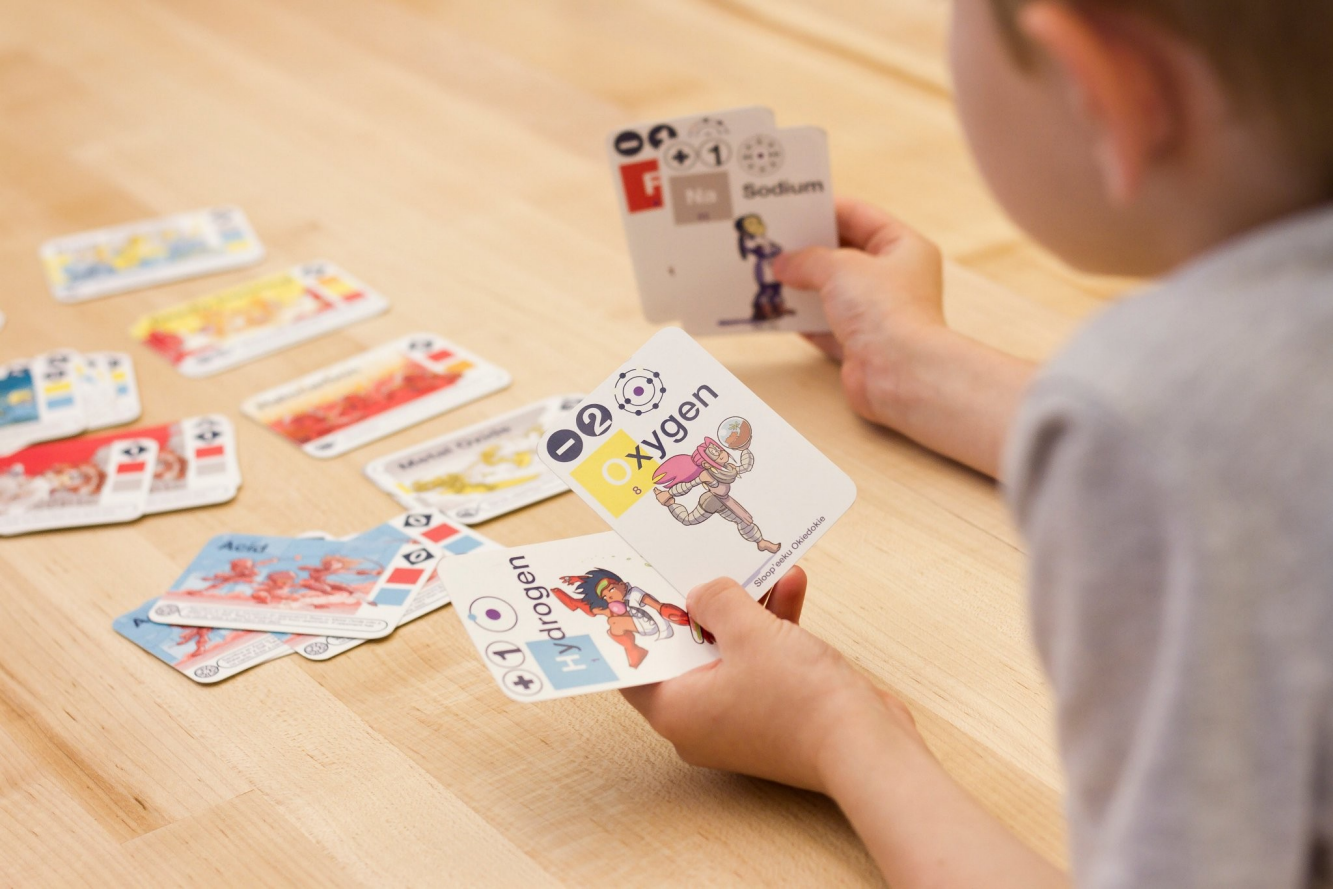
A schoolchild sits down to enjoy a game of *Science Ninjas: Valence*. *Image credit*: *Agustina Isidori*.

An example of an analogue science game that has really thought about its audience is *Science Ninjas*: *Valence* [27,28], an educational game designed to teach schoolchildren aged 8 to 12 about how molecules form from different elements. By creating a game that can be played in 10 to 25 minutes (and thus easily fit into a lesson or a lunch break), which takes very little time to explain, which can easily fit onto a classroom table (Fig 2), and with graphics designed to appeal to younger players, this game carefully considered the constraints of its intended audience.

The designers of *Science Ninjas*: *Valence* also considered the target audience (schoolchildren), creating a “Junior” version with simplified rules aimed at younger audiences.

### Rule 3: Adapt an existing game

If you are unsure of where to start, then we recommend that you begin by adapting a commercially available off-the-shelf (COTS) game. Doing so has several benefits: you know that the game works, that it is fun, and that it already has an established audience with whom you can interact. Adapting such a game (by creating an expansion or modifying the rules) will also help you in your development as a game designer, as you will have to learn how the mechanics of these COTS games work.

**Fig 3:**
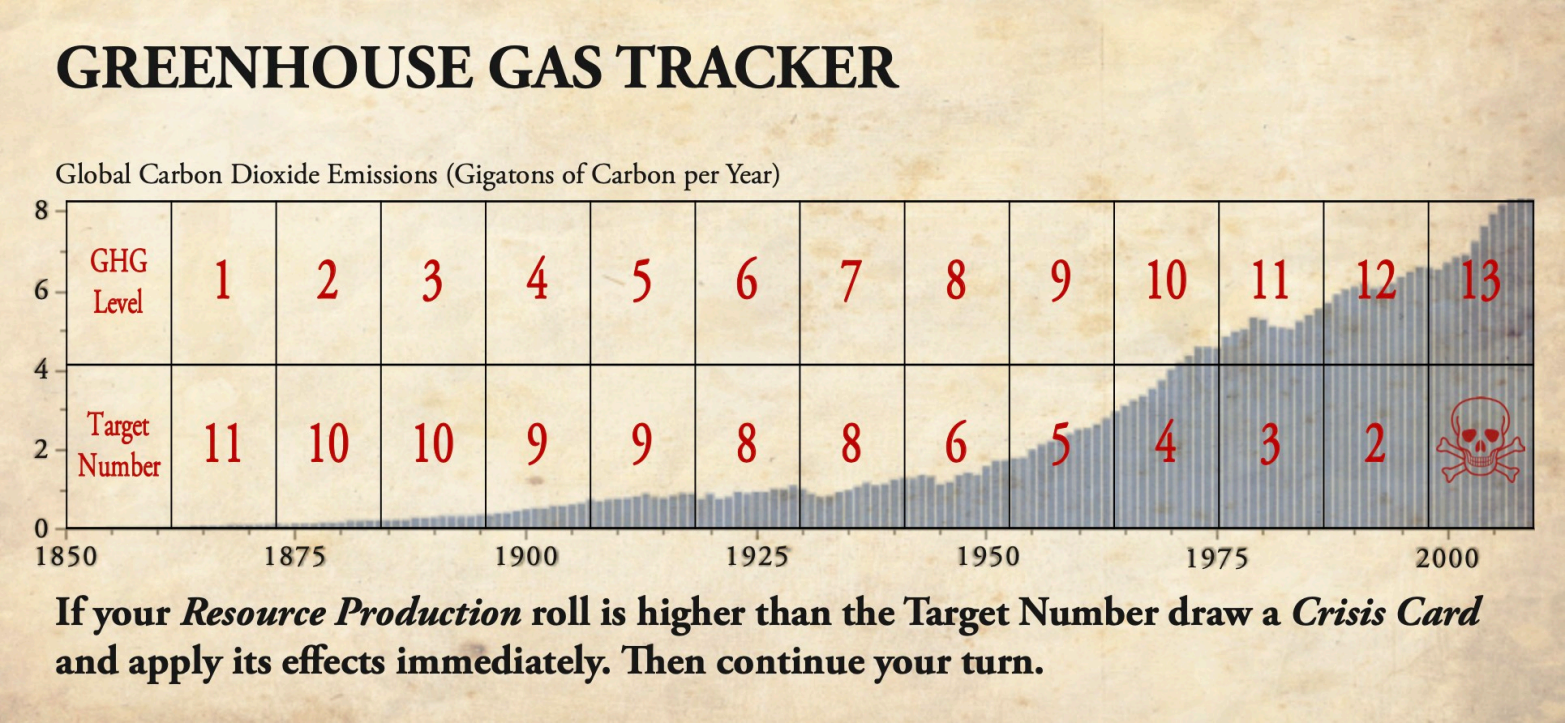
The Greenhouse Gas Tracker from the *Catan*: Global Warming scenario [29]. This scenario can be downloaded as a free print-and-play game in both English and German (https://www.pnparcade.com/products/catan-scenarios-global-warming). A copy of the original version of *Catan* is required to play.

For example, when we designed a “Global Warming” scenario [29] (Fig 3) for the popular game *Catan* [30,31], we aimed to create an experience for our audience (regular gamers) that enabled them to revisit a familiar game but which invited them to reconsider the game’s themes of “settlement” (national expansion and resourcing) in the context of runaway global warming effects. In doing so, we were also inspired by the work of others who had successfully adapted *Catan* to engage gamers on topics ranging from sustainability [32] to colonialism [33].

There are plenty of COTS games with readily identifiable “scientific” themes for inspiration. Such games include *Wingspan* [34] (a competitive deck-building game in which players compete to attract a diverse collection of birds to their wildlife reserves), *Pandemic* [35] (a cooperative action point allowance game in which players work to stop a viral pandemic), *Petrichor* [36] (a hand management game in which players compete to control the weather), and *Gut Check* [37] (an educational board game designed to explore the beneficial aspects of microbes) [38]. Similarly, there are many COTS games that could easily be adapted to develop dialogue around a range of scientific topics. Examples in the field of computational biology include *Photosynthesis* [39] (a strategy game in which players compete for sunlight to grow their trees), *Morels* [40] (a competitive card drafting game where players collect mushrooms), and *Pathogenesis* [41] (a competitive deck-building game in which players take the role of bacterial pathogens attacking the human body).

If you decide to modify a COTS game, then we recommend contacting the publisher of the game to check if there are any legal or copyright issues in what you are proposing.

### Rule 4: Use mechanics (not text) to convey messages

Games are well suited to the modelling of systems and behaviours and in encouraging players to interact with those systems [42]. Analogue games in particular offer players a direct engagement with the systems that they model via the requirement that they engage directly with the game’s operational rules (the rulebook) and the principles (often logical or mathematical) that underpin those rules. However, games are necessarily simplifications of complex realities, and while their design demands close attention to the scientific topic that you are aiming to explore or simulate (Rule 1), it will be necessary to identify the key ideas that you want to share.

**Fig 4:**
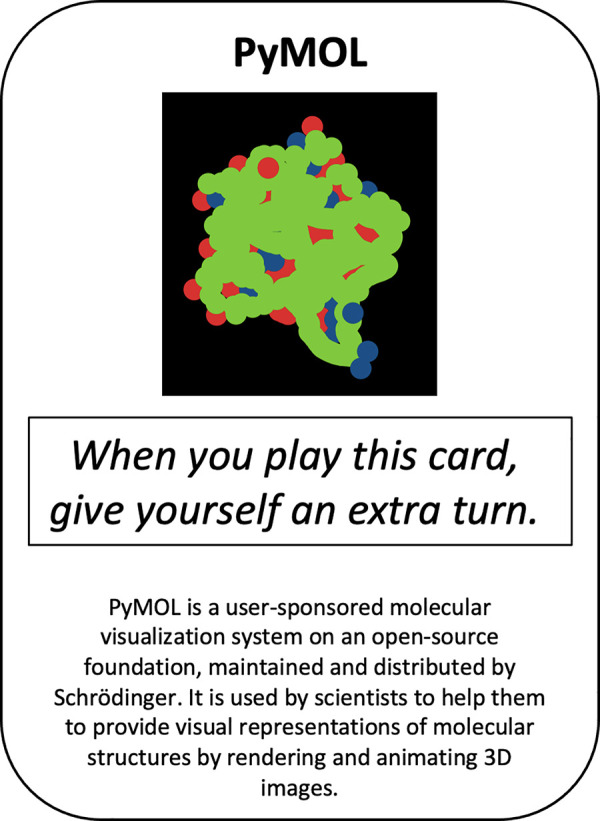
An early prototype for a game about developing a global map of the protein shape universe [43]. To read this “flavour” text, the player needs to temporarily remove themselves from the game experience.

A common mistake in the design of analogue science games is an overreliance on text. In our experience, players often ignore this “flavour” text, as they are far more interested in working out how they can play (and win) the game in hand. In Fig 4, the key information is the in-game effect (have an extra turn) which has no direct connection to the information about PyMOL. As such, this scientific information is at best superfluous and at worst intrusive.

Rather than text, then, consider how you can use the mechanics of the game itself to convey this information. For example, if you are designing a game about a global map of the protein shape universe [43], then, instead of having several different cards with lots of flavour text (that is unrelated to the ways in which the cards are used in the game), you might create a game in which players create different 3D shapes and assemblies of proteins. Such a game would likely give the players a clearer understanding of the processes that are being discussed, presenting them with a kinaesthetic experience for discovery.

There are many different types of game mechanics; indeed, the BoardGameGeek (www.boardgamegeek.com) website lists over 150 different game mechanics. We direct interested readers to *Building blocks of tabletop game design*: *An encyclopedia of mechanisms* [44] for a clear account of the majority of these mechanics and encourage you to think about which is most appropriate for your audience and your chosen scientific topic. For example, if your aim is to explore infectious disease forecasting [45], which might involve understanding how the behaviours of these diseases (and their underlying processes) evolve over time, then a “deck-building” mechanism (in which players construct their own decks of cards and aim to manage the order in which they are drawn) might be more appropriate than a “dexterity” mechanism (in which players flick or balance various game elements). An infectious disease deck builder would require players to understand the mechanistic models that their decks have created and manage statistical variants, both of which are essential for infectious disease forecasting. Similarly, thinking about the kind of interactions that you want your audience to have is vital. Competitive games might be suitable if you are setting out to simulate an inherently competitive concept (such as the competition-driven evolution of organismal complexity [46]). Likewise, collaborative games are great for promoting dialogue and have the advantage of allowing new players to get to grips with the rules through peer learning. Conversely, while “solo” games (those played by 1 person either against themselves, a set target, or a set of rules simulating another player—sometimes referred to as an artificial intelligence or AI player) might not seem like the most obvious way of establishing dialogue or communicating facts, designing a game with a solo variant is a great way of making games more accessible (Rule 6) and has particular application if you are considering audiences outside of mainstream education.

Finally, if you want to provide further scientific information for your players, then consider including this in a glossary or addendum (Rule 9). Doing so will allow those who are interested to find out more without having a negative impact on the gaming experience.

### Rule 5: Use artwork and graphic design to reinforce your message and improve gameplay

Getting the artwork and design of your game right is important for more than simply reasons of aesthetics. Visual learning is a type of learning style in which people prefer to use images (over reading, auditory, or kinaesthetic experiences) to communicate ideas and information [47]. It is an important method for enhancing learning and engaging interest [48] and has been shown to be effective at both disseminating facts and helping to enable dialogue [49]. Therefore, the design, style, and artwork of your game offer an opportunity to reinforce the way in which your scientific topic is communicated (Rule 1) to your intended audience (Rule 2).

**Fig 5:**
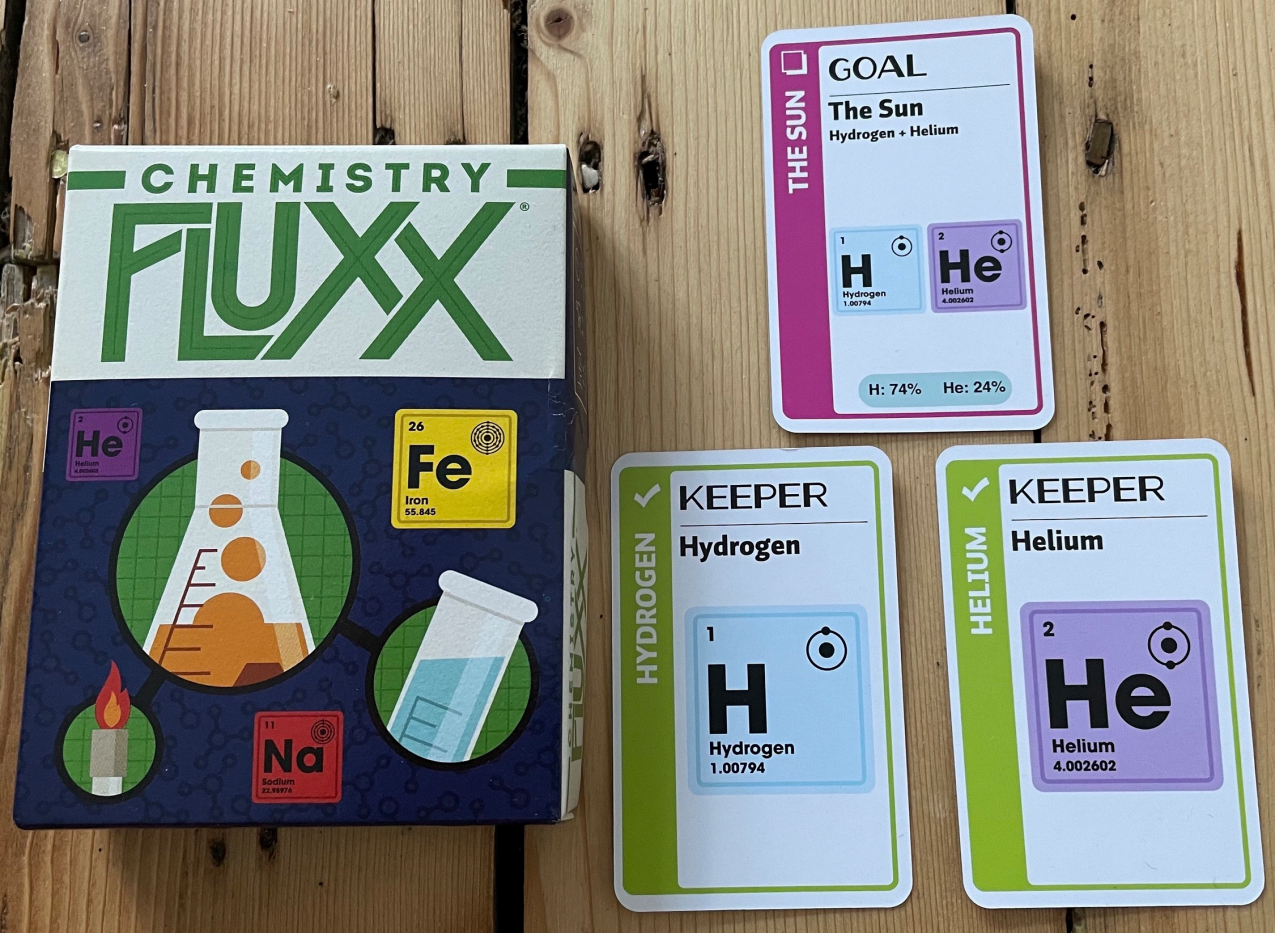
A selection of cards from the game *Chemistry Fluxx* [50]. In this setup, players win by achieving the “Goal” of having 2 “Keeper” cards in their hand (“Hydrogen” and “Helium”). The combination of text and graphic design, which recalls the periodic table, helps to convey information (that the sun is made up mostly of hydrogen and helium) in a clear and accessible manner that helps to engage interest through visual learning while also supporting gameplay (set collection/pattern matching).

An example of a COTS game that makes excellent use of artwork and design to support both gameplay and learning is the card game *Chemistry Fluxx* [50] (Fig 5). In this game, players draw and play cards, with endgame conditions being determined by specific “Goal” cards that are revealed during a game. As can be seen from Fig 5, these goals also include specific chemistry-based facts. Note that this is not superfluous flavour text (Rule 4) but rather a careful integration of game mechanics and theme, as players use cards from their hands to match the conditions set by these goals. The crisp design of the cards and use of images appeal to the visual learner and help to reinforce the topic (in this case, basic chemistry) that is being communicated.

### Rule 6: Make your game accessible

Once you have determined the needs and experiences of your intended audience (Rule 2), you should ensure that your game will be as accessible as possible. Accessibility should be considered at the very start of the design process and not as a box-ticking afterthought (this could easily have been Rule 1). When creating an analogue science game, this is especially important; you are aiming to make a scientific topic accessible to a diverse audience, many of whom will be nonexperts and any barriers to entry risk excluding participants, confusing facts, or curtailing dialogue.

**Fig 6:**
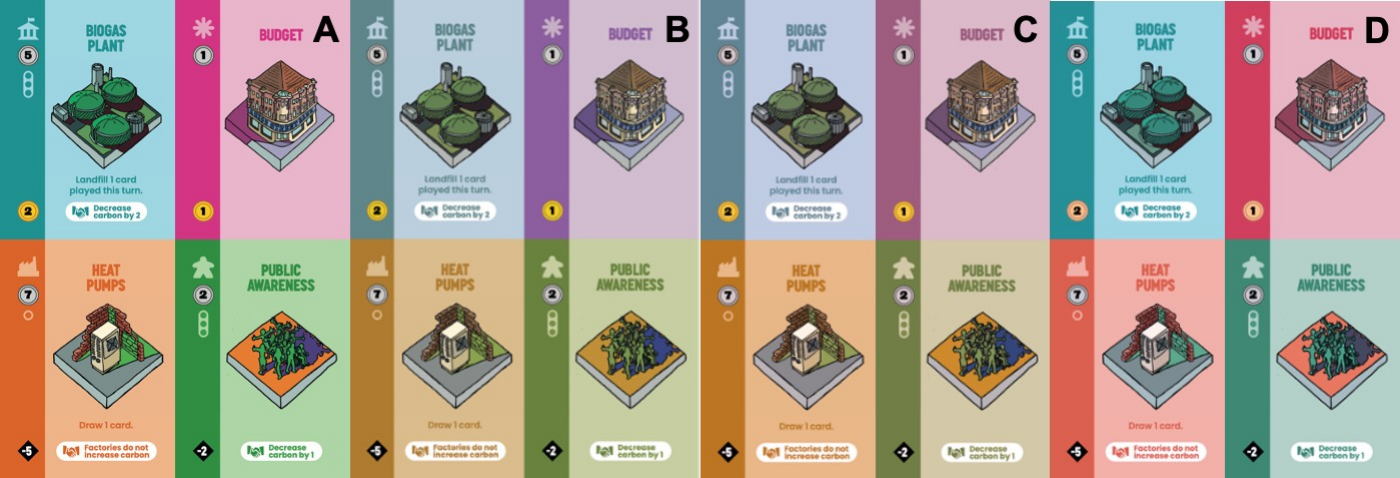
An image showing how a selection of cards from the game *Carbon City Zero: World Edition* [52] look to someone with no colour blindness **(A)**, anomalous trichromacy with red-weak protanomaly **(B)**, anomalous trichromacy with green-weak deuteranomaly **(C)**, and anomalous trichromacy with blue-weak deuteranomaly **(D)**. Introducing symbols that correspond to the different colours of the card can help to remove the reliance on colour, helping those people with visual impairments, and ultimately, making the game easier to play for everyone. This image was prepared using the free “Color Blindness Simulator” from the website www.color-blindness.com.

Making your game more accessible will ultimately make it more playable and provide a better experience for all. For example, if you make a game easier to play for people with visual impairments (Fig 6), then you also make it easier for any person to play in conditions of poor lighting or where space is limited (such as a crowded pub or a busy festival). This is what is known as the “curb cut effect,” i.e., when you make improvements for people who are living with disabilities, you make improvements for everybody else as well [51].

Games accessibility expert Michael Herron has written several articles on how to make analogue games more accessible [53,54] and runs a website (www.meeplelikeus.co.uk) which provides extremely useful advice on how and why to make your analogue game accessible. One of the best ways of making your game accessible is in learning from those games that do it well, and Herron’s “Accessibility Teardowns” of well-known games provide a useful resource in this respect. COTS games with science-related themes that are worth looking at in this regard include *NMBR 9* [55] (an abstract strategy game in which players must stack number-based cards on top of one another without leaving any gaps) and *Reef* [56] (a hand management game in which players take on the role of a coral reef, selecting colours and patterns in which to grow and expand). *NMBR 9* scores highly in terms of visual accessibility, relying on shape rather than colour to convey key information, while *Reef* uses tokens that have different shapes corresponding to the value and purpose of each token, which is a great aid to people living with blindness and vision loss.

### Rule 7: Make your game fun

Your game should be enjoyable. Ideally, people should want to play it of their own accord and not simply because it is part of an educational or learning experience. What is it that makes a game fun? Here are some aspects to consider:

#### Flow

Flow can be considered to be the feeling of complete focus in an activity, resulting in a high level of enjoyment and fulfilment [57]. To what extent does your game encourage flow? How does your design provide an immersive and rewarding experience for the player?

#### Challenge

Does your game present a challenge to your players? With your intended audience in mind, your game should be difficult enough so that it does not become boring, yet not so challenging that it becomes frustrating.

#### Skill versus Luck

How does your game treat the relationship between skill and luck? *Snakes and Ladders*, for example, is a game based entirely on luck, with your only actions determined by the random roll of a dice. A game like *Chess*, on the other hand, depends almost entirely on the skill of the player. Too much reliance on luck can leave players feeling detached from a game’s outcome. Conversely, a degree of luck can help level out differences in player skill and allows a wider range of players to engage in the same activity. The inclusion of random elements is also useful in developing activities where nonlinear progression and unpredictable outcomes are desired and can also have the effect of increasing the replayability of a game.

#### Competition

How competitive is your game? Is there the opportunity for players to win at any stage, or have you designed the game so that one player can build up an unassailable lead? You should try to avoid the latter scenario—keeping all players “in the game” for as long as possible will help them feel engaged.

**Fig 7:**
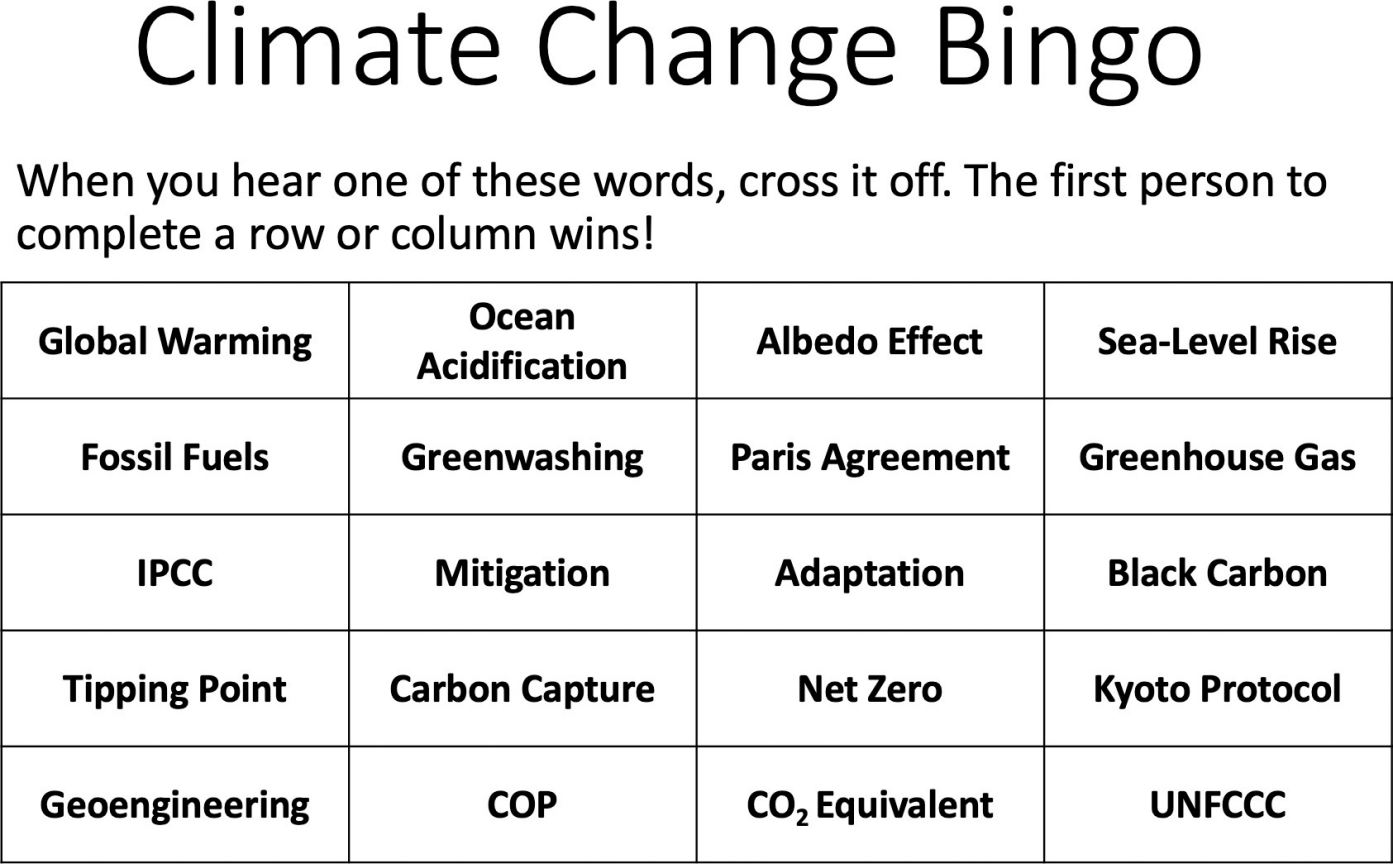
*Climate Change Bingo*. The fun of playing this game will largely be determined by the audience.

However, with all these aspects, the extent to which a game will or will not be fun to play largely depends upon the audience that will be playing it (Rule 2). Fig 7 shows a simple bingo variant designed to start conversations around climate change. For some audiences, this will be an engaging and enjoyable game to play while learning about a subject, whereas for other audiences, such a simple game might well miss the mark.

One of the best ways to determine what makes some games more enjoyable than others is to play lots of games yourself. Play a range of games with different game mechanics, different narrative elements, and different levels of complexity, and play them with different groups of people. The BoardGameGeek website is a treasure trove of information that will help you select games, including feedback from the gaming community about what they think constitutes a fun game. Having played several games, think about which ingredients make this game fun to play and what aspects you can borrow, and be inspired by, for your own game development.

Two specific games that we would recommend include *Pandemic* [35] (a cooperative game about global pandemics) and *Terraforming Mars* [58] (a hand management game in which players must compete to build a futuristic corporate empire on the red planet). These are both games that can be used to initiate dialogue about specific scientific topics but which have proved to be popular with a general gaming audience.

### Rule 8: Playtest all aspects of your game

Before you can use your game to convey facts or start conversations, you need to make sure that it works [59], and to do this, you need to playtest it [60]. There are several stages to playtesting, beginning with prototyping in which you sit down and play the game yourself (maybe several dozen, or several hundred, times) to make sure that it is balanced and doesn’t have any glaringly obvious barriers to play. Once you are confident that the game works (and that it is at least potentially fun to play), then you should give it to a few trusted colleagues or playtesters who can provide honest and critical feedback about your game. Once you have received feedback from this trusted “inner circle,” then it is time to open it up to a wider audience [60]. Playtesters may be recruited from BoardGameGeek or other such communities, via specific playtesting sessions that you organise, or at gaming events such as the UK Games Expo or Gen Con, many of which will have playtesting areas for games designers to test out their latest work.

In all these stages of playtesting, you are effectively asking your playtesters to provide a stress test on the game to see if it is broken, as well inviting them to offer information about the gaming experience. To make this as effective as possible, make sure that you provide your playtesters with the materials they need to provide the feedback that you need. For example, if you are trying to decide which tokens you should use to best represent different microbes for a game about invasion and colonisation resistance in microbial communities [61], then be sure to give your playtesters a range of tokens and ask them to feedback on which ones they thought were most effective and why. At least one of your playtesting sessions should involve an “unaided rules test” in which playtesters are given your game and rules without you (the games designer) being there to answer any queries. This is an essential step in helping to ensure that your game can be played, and to make this stage of playtesting most effective, we recommend running it with the audience for which your game was designed (Rule 2).

**Fig 8:**
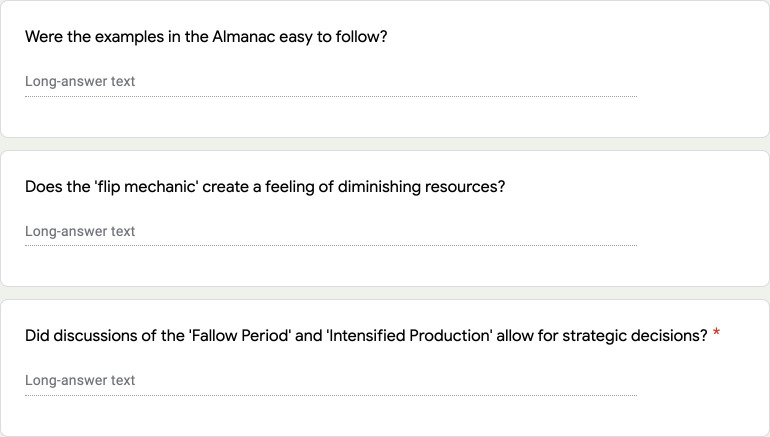
A section of a survey used to gather feedback during the playtesting of *Catan*: Global Warming. This section was designed to ask specific questions about the ease of following the rules, the scientific accuracy of the game, and the capacity for dialogue.

While the above advice is true of all analogue game design, when designing an analogue science game, there are two additional aspects that need to be tested. First, is the science that is represented in your game correct? And second, how successful is it in communicating facts or generating dialogue (depending on what type of game you are trying to design (Rule 1))? To test the scientific accuracy of the game, it is necessary to playtest it with experts working in the field to ensure that it is not conveying information that is incorrect or misleading. Similarly, given the purpose of the game (communicating facts/generating dialogue), it will be necessary to run playtests with your intended audience. Moreover, if you wish to assess the impact of the game, then you should consider how you will capture this via qualitative feedback, for example, through short surveys, debriefs, or recordings (with permissions) of the sessions themselves (see Fig 8). In summary, when playtesting your analogue science game, you need to check for scientific accuracy, audience fit, and the capacity for the game to disseminate accurate facts or start meaningful conversations.

At every stage of the playtesting experience, take on board the advice of your playtesters but don’t feel that you need to modify your game to accommodate every comment. Think of this feedback as an analogue of the peer review that you receive when submitting a manuscript to a scientific journal. You don’t have to make all the requests that the reviewer is asking of you, but you should be able to defend your decision not to [62].

Finally, remember to be respectful of your playtesters and to acknowledge their efforts and guidance. This usually takes the form of crediting any playtesters in your game’s rulebook.

### Rule 9: Supplement your game

**Fig 9:**
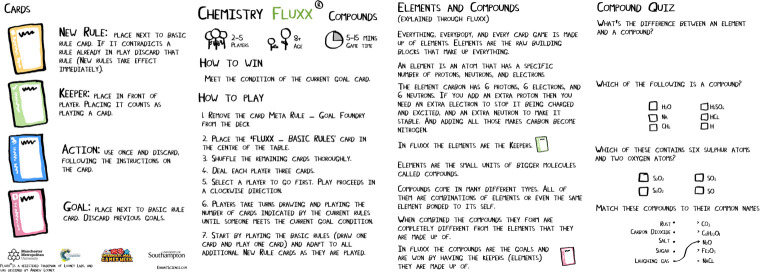
Learning materials that were designed for the game *Chemistry Fluxx* [50]. These resources provide additional information for how the game might be used by young children to discuss the differences between compounds and elements. These resources were produced in collaboration with the biochemist and cartoonist Matthew Partridge [64] and the librarian Darren Edwards, as part of a grant from the Royal Society of Chemistry.

In Rule 4, we recommended that any additional scientific information might be best included as a glossary or addendum to the game. While your game should be designed to be played without the need for such additional information, by considering the needs of your intended audience (Rule 2) and how and where they might be playing your game, you can potentially add to their experience and a debrief session following a game can be extremely useful in supporting game-based learning [63]. For example, if you are designing a game that aims to convey the difference between an electron and a compound to a group of younger schoolchildren, then you might consider providing additional materials in the form of simple diagrams or quizzes (Fig 9) to help reinforce this learning.

In developing these additional materials, remember to consider the limitations and constraints that may be imposed by both your target audience and the spaces in which they are likely to encounter the game. Furthermore, we recommend that you provide links to scientific research articles and other resources where interested parties can find out further information about the scientific topic in question.

### Rule 10: Collaborate and share

The final point that we would like to stress is the importance of collaboration. In designing any analogue game, it is necessary to work with a team of people, each of whom brings their own unique skills. It is highly unlikely that any one individual possesses the art, graphic design, and game design skills that are needed to make an analogue game.

This is crucial in the design of analogue science games, for which specific scientific expertise is also required. However, rather than being seen as a barrier, this should be seen as an opportunity. As scientists, we regularly collaborate with other scientists to answer specific scientific hypotheses, and in doing so, we can pool our various resources (both intellectual and physical) to produce the most optimal solution(s). Game design is the same, and working with trained artists, graphic designers, and game designers will result in a much better game while significantly reducing the workload for the individuals involved.

Indeed, having read these 9 rules above, you might conclude that you yourself are not the person to take on some of the design work itself. Instead, you might contribute expertise in the initial stages, identifying the topic (Rule 1) and the audience (Rule 2), before later contributing towards playtesting the scientific accuracy (Rule 8) and developing the supplementary materials (Rule 9). On a practical note, in designing your game, you should be conscious of the fact that you might only be able to get so far without commissioning a professional artist and/or graphic designer and it might be necessary to factor in time and other resources to cover this work.

We would also encourage designers to consider the idea that these collaborations might continue beyond the initial creation of the game. One of the greatest benefits of designing analogue games is that they can be easily and quickly adapted by their players (especially when compared to video games). Anyone with a pen or pencil has the capacity to retheme, remix, and adapt a COTS game in a way that is just not possible with video games (Rule 3). By encouraging players to create house rules, change win conditions, and even design their own expansions for your game, you open up the possibility for the dialogue to continue post publication.

To encourage the adaption of your game, you might consider releasing it under a Creative Commons CC BY NC SA 4.0 license, thereby allowing others to remix and adapt your game noncommercially, providing they credit you and license their own creations under identical terms. Similarly, you could release the assets of the game (artwork, rules, etc.) under a similar license and encourage others to use them to design their own analogue science games.

**Fig 10:**
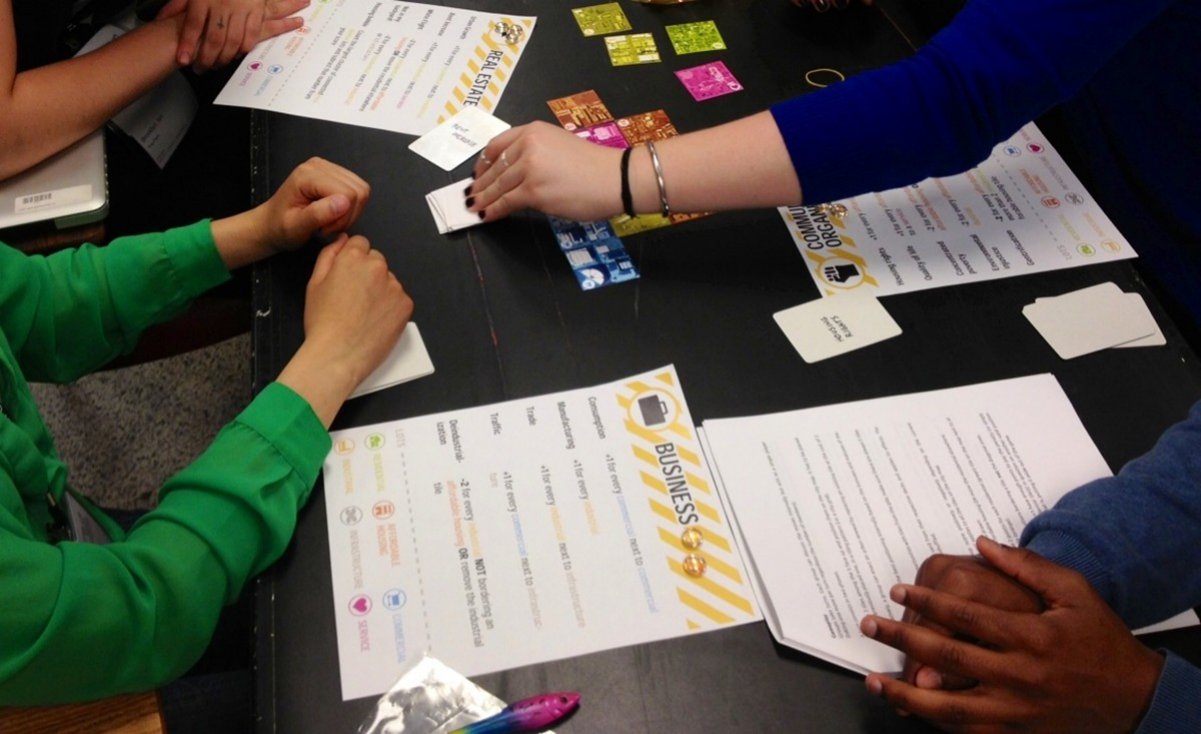
A group of players collaborate to adapt *MultipliCITY* for their own target audiences. *Image credit*: *Molleindustria 2014; CC BY 4*.*0*.

An example of a game designed to engender collaboration in this way is *MultipliCITY* (Fig 10) [65,66], a board game about city planning that was developed by Moleindustria to be discussed, extended, and modified in a workshop context environment. This game was released under a CC BY 4.0 license and comes complete with ideas for how players and game designers might collaborate to adapt the basic mechanics of a game. It can be downloaded from www.molleindustria.org/blog/multiplicity, and it is a good example of how building future adaptation into games can support their long-term development, often leading to their use in ways that their designers might never have originally imagined.

If you decide that you want to publish your game via a game publisher, or a crowdfunding platform such as Kickstarter (www.kickstarter.com), then we recommend that you still make a “print and play” (PNP) version of your game available (ideally without charge). Doing so will make your game accessible to a wider audience and will encourage others to adapt your game, often in ways that you could never have imagined. PNP Arcade (www.pnparcade.com) provides one such platform where game designers can upload PNP versions of their games for others to download, and, again, BoardGameGeek is a great resource for both finding and distributing PNP versions of games.

## Conclusions

Analogue games present an effective and immersive means by which to engender meaningful dialogue. Designing a game that enables players to have this dialogue about a scientific topic, while also enjoying the game and becoming immersed in the experience, is a challenging but ultimately rewarding experience. We hope that these “rules” have provided a useful introduction into game design and what it takes to make a “good” analogue science game. If we were to summarise all 10 rules into one pithy recommendation, it would be this: play more games, more often, and with more people.
